# *CYP17*, *GSTP1*, *PON1 *and *GLO1 *gene polymorphisms as risk factors for breast cancer: an Italian case-control study

**DOI:** 10.1186/1471-2407-9-115

**Published:** 2009-04-20

**Authors:** Cinzia Antognelli, Chiara Del Buono, Vienna Ludovini, Stefania Gori, Vincenzo N Talesa, Lucio Crinò, Francesco Barberini, Antonio Rulli

**Affiliations:** 1 Department of Experimental Medicine, University of Perugia, Perugia, Italy; 2 Medical Oncology Division, Azienda Ospedaliera of Perugia, Perugia, Italy; 3 Breast Unit, Department of Surgical, University of Perugia, Perugia, Italy

## Abstract

**Background:**

Estrogens, environmental chemicals with carcinogenic potential, as well as oxidative and carbonyl stresses play a very important role in breast cancer (BC) genesis and progression. Therefore, polymorphisms of genes encoding enzymes involved in estrogen biosynthesis pathway and in the metabolic activation of pro-carcinogens to genotoxic intermediates, such as cytochrome P450C17α (CYP17), endogenous free-radical scavenging systems, such as glutathione S-transferase (GSTP1) and paraoxonase 1 (PON1), and anti-glycation defenses, such as glyoxalase I (GLO1), could influence individual susceptibility to BC. In the present case-control study, we investigated the possible association of CYP17 A1A2, GSTP1 ILE105VAL, PON1 Q192R or L55M, and GLO1 A111E polymorphisms with the risk of BC.

**Methods:**

The above-said five polymorphisms were characterized in 547 patients with BC and in 544 healthy controls by PCR/RFLP methods, using DNA from whole blood. To estimate the relative risks, Odds ratios and 95% confidence intervals were calculated using unconditional logistic regression after adjusting for the known risk factors for BC.

**Results:**

CYP17 polymorphism had no major effect in BC proneness in the overall population. However, it modified the risk of BC for certain subgroups of patients. In particular, among premenopausal women with the A1A1 genotype, a protective effect of later age at menarche and parity was observed. As to GSTP1 and PON1 192 polymorphisms, the mutant Val and R alleles, respectively, were associated with a decreased risk of developing BC, while polymorphisms in PON1 55 and GLO1 were associated with an increased risk of this neoplasia. However, these findings, while nominally significant, did not withstand correction for multiple testing.

**Conclusion:**

Genetic polymorphisms in biotransformation enzymes CYP17, GSTP1, PON1 and GLO1 could be associated with the risk for BC. Although significances did not withstand correction for multiple testing, the results of our exploratory analysis warrant further studies on the above mentioned genes and BC.

## Background

Breast cancer (BC) is both the prevailing malignancy and the most common cause of cancer death among women in Western countries [1]. Estrogens, dietary factors, life-style, environmental chemicals with carcinogenic potential, as well as oxidative and carbonyl stresses, play a very important role in BC pathogenesis and progression [2-8]. It is, therefore, plausible that polymorphisms of genes encoding enzymes involved in estrogen biosynthesis pathway or metabolic activation of pro-carcinogens to genotoxic intermediates, endogenous free-radical scavenging systems and anti-glycation defenses, may influence individual susceptibility to BC.

Much interest has long been addressed to cytochrome P450c17α (CYP17) and glutathione S-transferase 1 (GSTP1) genes, encoding enzymes involved in estrogen biosynthesis and metabolism [9] or in environmental carcinogens detoxification and exo- and endogenous xenobiotic transformation [10], respectively. Despite some studies have confirmed a link between CYP17 [11-14] or GSTP1 [15-18] gene polymorphisms and the risk of BC, others have failed to find such an association [19-27] and conflicting results have been obtained. Hence, the clinical relevance of these polymorphic genes remains to be fully elucidated and needs further investigation.

Moreover, the search for additional metabolizing polymorphic genes as potential susceptibility factors in BC, is needed. In particular, paraoxonase 1 gene (PON1), encodes a serum high-density lipoproteins (HDL)-associated enzyme [28], playing an important role in lipid metabolism as an antioxidant molecule, through (a) hydrolyzation of active oxidized phospholipids, (b) destruction of lipid hydroperoxides and H_2_O_2 _(peroxidase-like activity), (c) preservation of HDL integrity and function and, finally, (d) prevention of LDL oxidation. Additionally, paraoxonase 1 is consistently implicated in the elimination of carcinogenic lipid-soluble radicals from lipid peroxidation [29]. Therefore, it is reasonable to expect that the activity of such an enzyme may influence BC development. In particular, loss of the paraoxonase 1 care-take function could play an important role in increasing the breast vulnerability to genomic damage caused by inflammatory oxidants, dietary carcinogens, as well as in estrogen-lipidic metabolism, that may modulate the progression of breast tumor. The main paraoxonase-encoding gene, PON1, is polymorphic in human populations and the expression of such a gene widely varies in human populations [30]. Two common single-base mutation polymorphisms (SNP) have been described in the encoding region of human PON1, which lead to glutamine → arginine substitution at 192 (Q192R) or a leucine → methionine substitution at 55 (L55M). Both these polymorphisms influence PON1 activity [31,32]. So far, very few information is available about the association of PON1/192 and 55 polymorphisms with BC risk and only in selected populations [33,34]. It was, therefore, aim of the present study also to analyze the relationships of PON1 allelic variants with the risk of BC and to clarify the question whether both gene variations might be useful genetic markers of breast tumor.

Glyoxalase I (GLO1) gene encodes an anti-glycation defence enzyme that decreases the concentration of dicarbonyl compounds (alpha-oxoaldehydes), such as methylglyoxal (MG), the most reactive glycation precursor [35]. Since substantial evidence exists on the role of carbonyl stress and DNA/protein glycation in relation to BC pathogenesis [7,8], it is possible that allele variants in GLO1 gene may predispose to the risk of developing BC and affect the course of the disease. A single nucleotide polymorphism in GLO1 gene, C419A, causing an Ala111Glu (A111E) change in the protein sequence, has been recently identified [36]. Therefore, since there are no existing data in this connection, we finally evaluated the distribution of GLO1 A111E polymorphism among BC patients as well as controls, to point out a possible association with the risk of BC.

We would also like to highlight that all these four genes have been simultaneously considered in the present study because all related to the control of oxidative stress. In fact, they encode for enzymes that detoxify free radicals or reduce potential substrates for their production. As well known, there is evidence that oxidative stress, resulting from either an excess of reactive oxygen species (ROS) or a deficiency in antioxidant capabilities, may play a role in the etiology of BC [4,37]. In particular, CYP17 is responsible for catecholestrogens (CEs) formation via estrogen biosynthesis [38]. CEs can be oxidized to the corresponding ortho-quinone derivatives with concomitant formation of ROS [39]. From this aspect, estrogens have been proposed to trigger BC development via an initiating mechanism involving CEs [38]. GSTP1 and PON1 encode enzymes that carry out well known cellular mechanisms of antioxidant defence, playing a key role in the removal or detoxification of ROS, which is essential for preventing oxidative damage [40]. Finally, GLO1 encodes an enzyme that removes methylglyoxal (MG), a potent oxidative stress precursor. Free radical generation given by MG involves ROS, reactive nitrogen species (RNS), as well as organic radicals like MG radial or cross-linked protein radicals [41].

## Methods

### Patients

The study protocol followed the guidelines of our local ethics committee and the investigation was conducted with the ethical requirements defined in the Helsinki Declaration. All patients gave their informed consent to participate in the study.

Caucasian Italian women with a clinical and histological diagnosis of breast cancer (BC) (ductal carcinoma, 78.6%; lobular carcinoma, 15.9%; papilla tubular carcinoma, 1.3%; other, 4.2%) were enrolled in this study between June 2003 and July 2007 from the Breast Unit, Surgical Department of the University of Perugia, Umbria, Italy. Women with BC were identified via the Regional Cancer Registry, to which reporting of all malignant tumors is mandatory, and contacted via their doctors. In total, 547 women agreed to participate in the study, representing 89% of those who were contacted and found to be eligible. Histological staging of breast carcinoma was performed according to the current classification of the International Union Against Cancer [42]. Women showing axillary lymph node-positive disease (n = 190) or metastatic BC (stage IV; n = 19) at diagnosis were considered "advanced" cases. Women diagnosed with a tumor confined to the breast, either *in situ *(n = 46) or invasive (n = 292), were designated as "local". The age at diagnosis of BC was 55.3 ± 10.5 (SD) years and the patients had no previous history of BC or any other cancer. Control women (n = 544) were healthy Caucasian Italian women as well, randomly selected from the Umbrian Registry of Total Population, matched by age (55.0 ± 10.2 years; P = 0.11). The participation rate among controls was 87%. All these subjects were asked to complete a self-administered questionnaire on demographic factors, anthropometric measures, menstrual, reproductive, and breast feeding histories, use of contraceptives and exogenous hormones, medical and screening histories, first-degree family history of BC, occupational exposure, smoking history and alcohol consumption. All subjects were asked to provide a blood sample, to be used for genotyping. The median time between diagnosis and interview for BC cases was 2 months.

### Primers and Polymerase Chain Reaction (PCR)

Genomic DNA was extracted from heparinized peripheral whole blood, using the QIAamp Blood Mini Kit (Qiagen, Milan, Italy). The nucleotide sequence of primers is shown in Table 1. PCR amplification was carried out in a final volume of 50 μl, containing 20 ng of DNA, 20 pmoles of each primer, 100 mM of each dNTP, 1.5 mM MgCl_2_, 10 mM Tris-HCl pH 8.3, 50 mM KCl, and 1 U of Taq DNA polymerase. DNA amplification was performed conventionally; samples were subjected to 35 cycles of amplification consisting of 40 sec. denaturation, 30 sec. annealing, and 30 sec. extension. The final extension step at 72°C was extended to 10 min. The annealing temperature was optimized for each primer set.

**Table 1:** Oligonucleotide primer sequences used for genotyping

Gene	Oligonucleotides	Sequence (5'- 3')	Expected amplicon size (bp)
CYP17	230F	CATTCGCACTTCTGGAGTC	459
	642R	GGCTCTTGGGGTACTTG	
GSTP1	P105F	ACCCCAGGGCTCTATGGGAA	176
	P105R	TGAGGGCACAAGAAGCCCCT	
PON1 192	PON192F	TATTGTTGCTGTGGGACCTGAG	99
	PON192R	CACGCTAAACCCAAATACATCTC	
PON1 55	PON55F	GAAGAGTGATGTATAGCCCCAG	170
	PON55R	TTTAATCCAGAGCTAATGAAAGCC	
GLO1	GLO1F	TCAGAGTGTGTGATTTCGTG	700
	GLO1R	CATGGTGAGATGGTAAGTGT	

### Genotyping

SNPs detection was based upon Restriction fragment length polymorphism (RFLP) analysis after PCR amplification. CYP17 gene maps to chromosome 10. A single T (A1 allele) to C (A2 allele) nucleotide substitution in the 5'-untranslated region of CYP17 creates a recognition site for the MspA1 restriction enzyme. GSTP1 gene has been mapped to a small region of chromosome 11. The region of genomic DNA flanking exon 5 of GSTP1 gene was amplified. The A to G substitution in GSTP1 leads to the creation of a recognition site for the Alw261 restriction enzyme. PON1 gene is located at chromosome 7. Single-base substitutions, which lead to a change of a glutamine residue into arginine at amino acid position 192, and a change of leucine to methionine affecting amino acid 55 of the paraoxonase protein encoded by human PON1, create recognition sites for Alw1 (PON1/192) and NlaIII (PON1/55), respectively. GLO1 gene maps to chromosome 6. A C to A substitution in GLO1 exon 4, which changes Ala111Glu in the encoded protein, leads to the loss of a recognition site for the SfaNI restriction enzyme. PCR products were, therefore, digested for 3 hr at 37°C using either MspA1, Alw261, Alw1, NlaIII, or SfaNI, respectively, and separated by electrophoresis in 12% polyacrylamide gels. The band patterns were developed by silver staining to identify the single base pair change. All genotyping was carried out by laboratory personnel blinded to case-control status of the samples, which included quality control samples for validation. Concordance for quality control samples was 100%.

### Statistical Analysis

The obtained results were analyzed with the MedCalc statistical package (MedCalc, Mariakerke, Belgium). The associations between CYP17, GSTP1, PON1, GLO1 alleles and breast cancer (BC) were evaluated using unconditional logistic regression. All Odds ratio (ORs) and 95% confidence intervals (CIs) were adjusted for potential modifying factors, including age at menarche (continuous, log transformed), age at first full-term pregnancy (FFTP) (continuous, log transformed), history of benign breast disease (yes, no), first-degree family history of breast cancer (yes, no), waist-hip ratio (WHR) (continuous, log transformed), use of oral contraceptive (yes, no), alcohol consumption (ever, never), smoking habits (ever, never), level of education (low, medium/high) and postmenopausal use of estrogens (ever, never). Covariates as body mass index (BMI), breast cysts and physical activity were not included in the logistic model, because they did not alter the OR by > 5%, either separately or in combination. Since risk factors for BC significantly vary depending on menopausal status, we performed our analysis not only in the overall population, but also in premenopausal and postmenopausal subsets of women. Women who reported natural menopause or had undergone bilateral oophorectomy were classified as postmenopausal. Hysterectomized women with intact ovaries/ovary and women for whom the details of the operation were unknown were also classified postmenopausal, if they were no longer menstruating and were older than 50 years (median for menopause in Italian women). All of the others were classified premenopausal. WHR, BMI, and age at FFTP were dichotomized on the basis of the median values for population controls. Estimates of statistical significance were calculated by standard χ^2 ^analysis, or by Fisher's exact test, where cell numbers were < 5. We used Bonferroni correction to account for multiple comparisons, and a two-tailed P value < 0.000862 was considered statistically significant.

Descriptive analysis included determination of standard deviation (SD) for cases and controls as well as Student's t-test to evaluate differences between means. A two sided probability value of less than 0.05 was considered to indicate statistical significance. For each group (controls and cases), allele frequencies were calculated by direct gene counting. Deviations from Hardy-Weinberg equilibrium (HWE) were tested by χ^2 ^test.

## Results

### Patients characteristics

The main characteristics of the study subjects (cases, n = 547; controls, n = 544) and potential risk factors for breast cancer (BC) are shown in Table 2. The occurrence of premenopausal women was higher among population controls (42.3%) compared with BC patients (34.2%). The mean age at menarche [14.0 ± 1.54 (SD) years for controls, 14.2 ± 1.65 (SD) years for cases] and age at first term pregnancy [24.9 ± 4.15 (SD) years for controls, 25.2 ± 4.65 (SD) years for cases] were quite similar in both groups. First-degree family history of BC was associated with an increased risk of this malignancy (OR, 2.63; 95% CI, 1.60–4.40, P < 0.01). Quite weaker, but yet significant associations were observed for the history of benign breast disease (OR, 1.32; 95% CI, 1.02–1.72, P < 0.05) and WHRs over 0.91 (OR, 1.49; 95% CI, 1.17–1.91, P < 0.01). On the other hand, a significantly decreased risk was observed for women who had ever used oral contraceptives (OR, 0.55; 95% CI, 0.40–0.71, P < 0.01), for women with at least one child (OR, 0.60; 95% CI, 0.37–0.95, P < 0.01), and women aged 26–30 at FFTP (OR, 0.44; 95% CI, 0.30–0.65, P < 0.01). When tested for HWE, statistically significant deviations were detected between the observed and expected genotypic frequencies in all the tested genes, except CYP17.

**Table 2:** Selected characteristics of the study subjects and potential risk factors for breast cancer

	Cases (n = 547) (%)	Controls (n = 544) (%)	OR^b ^(95% CI)
	
Menopausal status			
Premenopausal	187 (34.2)	230 (42.3)	1.0
Postmenopausal	360 (65.8)	314 (57.7)	0.75 (0.44–1.29)
			
Age at menarche			
<13	99 (18.1)	87 (16.0)	1.0
13–14	230 (42.0)	212 (39.0)	0.73 (0.54–1.06)
≥ 15	218 (39.9)	245 (45.0)	0.91 (0.62–1.26)
			
Age at FFTP			
Nulliparous	116 (21.2)	65 (11.9)	1.0
≤ 25	269 (49.2)	296 (54.4)	0.76 (0.52–1.09)
26–30	107 (19.6)	138 (25.4)	0.44 (0.30–0.65)**
≥ 31	55 (10.0)	45 (8.3)	0.67 (0.38–1.12)
			
Number of full-term pregnancies			
Nulliparous	116 (21.2)	65 (12.0)	1.0
1	77 (14.1)	72 (13.2)	0.60 (0.37–0.95)**
2	160 (29.2)	204 (37.5)	0.45 (0.32–0.63)*
3+	194 (35.5)	203 (37.3)	0.55 (0.38–0.77)*
			
Use of oral contraceptives			
Never	359 (65.6)	275 (50.6)	1.0
Ever	188 (34.4)	269 (49.4)	0.55 (0.40–0.71)**
			
Postmenopausal use of estrogen			
Never	254 (70.6)	208 (66.2)	1.0
Ever	106 (29.4)	106 (33.8)	0.80 (0.60–1.02)
			
WHR			
≤ 0.91	216 (39.5)	270 (49.6)	1.0
> 0.91	331 (60.5)	274 (50.4)	1.49 (1.17–1.91)**
			
BMI (kg/m^2^)			
≤ 25.4	243 (44.4)	271 (49.8)	1.0
> 25.4	304 (55.6)	273 (50.2)	1.21 (0.96–1.55)
			
First-degree family history of breast cancer			
No	485 (88.7)	519 (95.4)	1.0
Yes	62 (11.3)	25 (4.6)	2.63 (1.60–4.40)**
			
History of benign breast disease			
No	340 (62.2)	375 (68.9)	1.0
Yes	207 (37.8)	169 (31.1)	1.32 (1.02–1.72)*
			
Education^a^			
Low	332 (60.7)	313 (57.5)	1.0
Medium	144 (26.3)	153 (28.1)	0.87 (0.66–1.15)
High	71 (13.0)	78 (14.4)	0.83 (0.57–1.25)
			
Current alcohol intake			
Never	309 (56.5)	272 (50.0)	1.0
Once a month or less	153 (28.0)	172 (31.6)	0.76 (0.57–1.03)
Daily-weekly	85 (15.5)	100 (18.4)	0.74 (0.51–1.04)
			
Smoking habits			
Nonsmokers^c^	415 (75.9)	396 (72.8)	1.0
Ex-smokers^d^	59 (10.8)	76 (14.0)	0.72 (0.49–1.07)
Current smokers^e^	73 (13.3)	72 (13.2)	0.95 (0.66–1.38)

^a^Low: none or primary school; medium: middle school; high: high school, trade school or university. ^b^Adjusted for age. OR: odds ratio; CI: confidencial intervals; FFTP: first full-term pregnancy; WHR: waist-hip ratio; BMI: body mass index. ^c^Nonsmokers = Never smokers; ^d^Ex-smokers = tobacco consumption stopped from 7.6 ± 4.2 years with a previous average tobacco consumption estimated at 47 pack-years (range, 20–106); ^e ^Current smokers = with an average tobacco consumption estimated at 43 pack-years (range, 20–200). *P < 0.05; **P < 0.01. The characteristics of participants with and without BC were compared by χ^2 ^test for categorical variables.

### CYP17 polymorphism and BC risk

The distribution of CYP17 genotypes and the ORs associated with BC are shown in Table 3. The A2 allele did not significantly affect BC risk, either in the overall population or when women were grouped according to menopausal status. No statistically significant differences were observed either when case patients were considered by tumor stage (Table 4). Conversely, we observed a protective effect of later age at menarche (≥ 13 years) among premenopausal women with the A1A1 genotype (OR, 0.28; 95% CI, 0.10–0.71, P for interaction = 0.11) and for premenopausal women with at least one child and the A1A1 genotype (OR, 0.26; 95% CI, 0.10–0.60, P for interaction = 0.12) (Table 5). In contrast, an increased risk of BC with borderline significance was observed for postmenopausal women with A1A2 or A2A2 genotype and BMI > 25,4 Kg/m^2 ^(Table 5).

**Table 3:** Associations between CYP17 genotype and breast cancer according to menopausal status

	A1A1	A1A2	A2A2	A1A2 and A2A2
	
All				
Cases, n (%)	229 (41.9)	258 (47.1)	60 (11.0)	318 (58.1)
Controls, n (%)	227 (41.7)	249 (45.8)	68 (12.5)	317 (58.3)
OR (95% CI)^a^	1.0	0.95 (0.72–1.24)	0.77 (0.51–1.20)^b^	0.90 (0.70–1.18)
Menopausal status at diagnosis				
Premenopausal				
Cases, n (%)	88 (47.1)	81 (43.3)	18 (9.6)	99 (52.9)
Controls, n (%)	100 (43.5)	99 (43.0)	31 (13.5)	130 (56.5)
OR (95% CI)^a^	1.0	0.88 (0.56–1.36)	0.66 (0.33–1.29)^b^	0.83 (0.55–1.25)
Postmenopausal				
Cases, n (%)	141 (39.2)	177 (49.2)	42 (11.6)	219 (60.8)
Controls, n (%)	127 (40.4)	150 (47.8)	37(11.8)	187 (59.5)
OR (95% CI)^a^	1.0	0.98 (0.68–1.41)	0.94 (0.53–1.68)^b^	0.97 (0.69–1.38)

^a^Adjusted for age, age at menarche, age at FFTP, number of full term pregnancies, first-degree family history of breast cancer, history of benign breast disease, use of oral contraceptives, WHR, alcohol consumption, smoking habits, level of education and postmenopausal use of estrogen. ^b^P for trend: all, 0.221; premenopausal, 0.389; postmenopausal, 0.954.

**Table 4:** Associations between CYP17 genotype and breast cancer according to menopausal status and tumor stage

	A1A1	A1A2	A2A2	A1A2 and A2A2
	
Premenopausal				
Controls, n (%)	100 (43.5)	99 (43.0)	31 (13.5)	130 (56.5)
Cases by stage				
Local, n (%)	48 (43.6)	50 (45.5)	12 (10.9)	62 (56.4)
OR (95% CI)^a^	1.0	1.02 (0.61–1.71)	0.76 (0.35–1.66)^b^	0.96 (0.58–1.56)
Advanced, n (%)	41 (53.2)	30 (39.0)	6 (7.8)	36 (46.8)
OR (95% CI)^a^	1.0	0.63 (0.35–1.25)	0.43 (0.16–1.16)^c^	0.57 (0.32–1.01)
Postmenopausal				
Controls, n (%)	127 (40.4)	150 (47.8)	37(11.8)	187 (59.6)
Cases by stage				
Local, n (%)	93 (40.8)	105 (46.0)	30 (13.2)	135 (59.2)
OR (95% CI)^a^	1.0	1.01 (0.65–1.52)	1.07 (0.57–2.16)^d^	1.02 (0.68–1.53)
Advanced, n (%)	47 (35.6)	73 (55.3)	12 (9.1)	85 (64.4)
OR (95% CI)^a^	1.0	0.98 (0.59–1.64)	0.87 (0.37–2.05)^e^	0.97 (0.62–1.53)

^a^Adjusted for age, age at menarche, age at FFTP, number of full term pregnancies, first-degree family history of breast cancer, history of benign breast disease, use of oral contraceptives, WHR, alcohol consumption, smoking habits, level of education and postmenopausal use of estrogen. ^b^P for trend = 0.591; ^c^P for trend = 0.061; ^d^P for trend = 0.835; ^e^P for trend = 0.829.

**Table 5:** Associations between CYP17 genotypes and breast cancer risk stratified by selected characteristics

	Premenopausal				Postmenopausal			
	
	A1A1		A1/A2 and A2A2		A1A1		A1/A2 and A2A2	
	Case/Control	OR^a ^(95% CI)	Case/Control	OR^a ^(95% CI)	Case/Control	OR^a ^(95% CI)	Case/Control	OR^a ^(95% CI)
Age at menarche								
< 13 yr	25/11	1.0	23/26	1.0	19/25	1.0	32/25	1.0
≥ 13 yr	64/89	0.28 (0.10–0.71)	75/104	0.77 (0.39–1.53)	119/103	1.31 (0.63–2.72)	190/161	0.84 (0.44–1.61)
Postmenopausal use of estrogen								
Never					100/89	1.0	154/119	1.0
Ever					39/40	1.15 (0.63–2.12)	67/66	0.86 (0.52–1.41)
Use of oral contraceptives								
Never	28/27	1.0	33/32	1.0	106/86	1.0	192/130	1.0
Ever	64/74	1.01 (0.45–2.27)	62/97	0.55 (0.29–1.03)	32/41	0.83 (0.41–1.74)	30/57	0.52 (0.29–0.98)
Parity								
Nulliparous	21/9	1.0	14/15	1.0	30/16	1.0	51/25	1.0
Parous	68/91	0.26 (0.10–0.60)	84/115	0.77 (0.32–1.94)	109/109	0.53 (0.25–1.08)	170/164	0.97 (0.36–1.17)
Age at FFTP for parous women								
≤ 25 yr	48/47	1.0	51/66	1.0	65/64	1.0	105/119	1.0
> 25 yr	22/35	0.83 (0.45–1.55)	33/43	1.34 (0.61–2.89)	43/42	0.92 (0.53–1.65)	64/63	0.59 (0.28–1.47)
BMI								
≤ 25,4	63/67	1.0	51/76	1.0	53/51	1.0	76/77	1.0
> 25,4	28/35	0.60 (0.27–1.34)	45/52	1.05 (0.57–1.93)	86/76	1.43 (0.75–2.72)	145/110	1.64 (0.97–2.78)
WHR								
< 0,91	41/58	1.0	42/72	1.0	47/58	1.0	86/82	1.0
≥ 0,91	47/40	1.61 (0.83–3.17)	57/60	1.52 (0.85–2.75)	91/68	1.68 (0.95–2.99)	136/106	1.12 (0.70–1.97)

^a^Adjusted for age, age at menarche, age at FFTP, number of full term pregnancies, first-degree family history of breast cancer, history of benign breast disease, use of oral contraceptives, WHR, alcohol consumption, smoking habits, level of education and postmenopausal use of estrogen.

### GSTP1 polymorphism and BC risk

The distribution of GSTP1 genotypes and the ORs associated with BC are shown in Table 6. We found that the frequency of the Val allele was significantly lower in the BC population than in the control group; 42% of the cases and 76% of controls carried at least one Val allele. Patients with the Ile/Val genotype and Val/Val genotype had a significant decrease for BC risk compared with the Ile/Ile genotype. Results were similar in pre- (46% of cases and 75% of controls were carriers of at least one Val allele) and postmenopausal (40% of cases and 78% of controls were carriers of at least one Val allele) women. No significant association was found between GSTP1 polymorphism and BC risk, according to tumor stage or potential hormone-related BC risk factors (age at menarche, postmenopausal use of estrogens, use of oral contraceptives, parity, age at FFTP, BMI or WHR) (data not shown).

**Table 6:** Associations between GSTP1 genotype and breast cancer

	Ile/Ile	Ile/Val	Val/Val	Ile/Val and Val/Val
	
All				
Cases, n (%)	315 (57.6)	217 (39.7)	15 (2.7)	232 (42.4)
Controls, n (%)	128 (23.5)	340 (62.5)	76 (14.0)	416 (76.5)
OR (95% CI)^a^	1.0	0.22 (0.14–0.30)	0.04 (0.01–0.09)^b^	0.15 (0.07–0.25)
Menopausal status at diagnosis				
Premenopausal				
Cases, n (%)	101 (54.0)	76 (40.6)	10 (5.4)	86 (46.0)
Controls, n (%)	58 (25.2)	140 (60.9)	32 (13.9)	172 (74.8)
OR (95% CI)^a^	1.0	0.27 (0.17–0.38)	0.14 (0.06–0.22)^b^	0.23 (0.16–0.35)
Postmenopausal				
Cases, n (%)	214 (59.4)	141 (39.2)	5 (1.4)	146 (40.6)
Controls, n (%)	70 (22.3)	200 (63.7)	44 (14.0)	244 (77.7)
OR (95% CI)^a^	1.0	0.23 (0.14–0.35)	0.09 (0.02–0.10)^b^	0.36 (0.24–0.55)

^a^Adjusted for age, age at menarche, age at FFTP, number of full term pregnancies, first-degree family history of breast cancer, history of benign breast disease, use of oral contraceptives, WHR, alcohol consumption, smoking habits, level of education and postmenopausal use of estrogen. ^b^P for trend = 0.001.

### PON1 polymorphisms and BC risk

The associations between the Q192R or L55M polymorphisms and BC risk are shown in Table 7. As to PON1 Q192R, 11.5% of cases and 37.5% of controls were carriers of at least one R allele. QR heterozygotes and mutant RR homozygotes had a lower risk of the breast disease compared to the QQ homozygotes. In particular, there was a trend in decreasing the risk with the number of R alleles. Results stratified by menopausal status at diagnosis, confirmed the OR of the general analysis only in the case of postmenopausal group, where the association was even more pronounced. In contrast, no significant association was observed in the premenopausal group.

**Table 7:** Association of genotypes for the PON1 Q192R and PON1 L55M SNPs and breast cancer

	QQ	QR	RR	QR and RR
	
PON1 Q192R				
All				
Cases, n (%)	484 (88.5)	50 (9.1)	13 (2.4)	63 (11.5)
Controls, n (%)	340 (62.5)	152 (28.0)	52 (9.5)	204 (37.5)
OR (95% CI)^a^	1.0	0.58 (0.32–0.99)	0.45 (0.18–0.88)^b^	0.55 (0.30–0.95)
Menopausal status at diagnosis				
Premenopausal				
Cases, n (%)	135 (72.2)	41 (21.9)	11 (5.9)	52 (27.8)
Controls, n (%)	144 (62.6)	64 (27.8)	22 (9.6)	86 (37.4)
OR (95% CI) ^a^	1.0	0.73 (0.51–1.43)	0.64 (0.40–1.44)^b^	0.68 (0.47–1.47)
Postmenopausal				
Cases, n (%)	349 (96.9)	9 (2.5)	2 (0.6)	11 (3.1)
Controls, n (%)	196 (62.4)	88 (28.0)	30 (9.6)	118 (37.6)
OR (95% CI) ^a^	1.0	0.12 (0.01–0.33)	0.08 (0.01–0.14)^b^	0.05 (0.02–0.12)
	
PON1 L55M	LL	LM	MM	LM and MM
	
All				
Cases, n (%)	107 (19.6)	115 (21.0)	325 (59.4)	440 (80.4)
Controls, n (%)	188 (34.6)	125 (23.0)	231 (42.4)	356 (65.4)
OR (95% CI)^a^	1.0	1.80 (1.36–2.56)	2.81 (1.95–3.64)^c^	2.42 (1.73–2.99)
Menopausal status at diagnosis				
Premenopausal				
Cases, n (%)	30 (16.1)	33 (17.6)	124 (66.3)	157 (83.9)
Controls, n (%)	78 (33.9)	70 (30.4)	82 (35.7)	152 (66.1)
OR (95% CI) ^a^	1.0	1.26 (0.66–2.39)	3.83 (2.15–5.87)^c^	2.67 (1.62–4.15)
Postmenopausal				
Cases, n (%)	77 (21.4)	82 (22.8)	201(55.8)	283 (78.6)
Controls, n (%)	110 (35.0)	55 (17.5)	149 (47.5)	204 (65.0)
OR (95% CI) ^a^	1.0	2.23 (1.39–3.40)	2.06 (1.30–2.97)^c^	2.59 (1.35–3.06)

^a^Adjusted for age, age at menarche, age at FFTP, number of full term pregnancies, first-degree family history of breast cancer, history of benign breast disease, use of oral contraceptives, WHR, alcohol consumption, smoking habits, level of education and postmenopausal use of estrogen. ^b^P for trend: all, 0.01; premenopausal, 0.29; postmenopausal, 0.001. ^c^P for trend: all, premenopausal, postmenopausal, 0.001.

Concerning PON1 L55M, this polymorphism was associated with a significantly increased risk of BC. Eighty percent of cases and 65% of controls were carriers of at least one M allele. When stratified by menopausal status, the frequency of the M allele was higher among both premenopausal (84%) and postmenopausal (79%) cases compared with the respective frequencies (66% and 65%) in the control subjects. Premenopausal women with the MM genotype had a significant increase for BC risk compared with the LL genotype. Postmenopausal women with the LM and MM genotype had a significant increase for BC risk compared with the LL genotype. PON1 Q192R and L55M genotypes were then analyzed according to tumor stage (Table 8). As to PON1 Q192R in the premenopausal group, such a genotype was associated with a lower risk of only advanced BC, compared with controls, while no significant correlation was found for local BC. In particular, premenopausal patients having advanced disease were less frequently carriers of the R allele (16%) compared with controls (37%). Heterozygotes QR and homozygotes RR had a significant decrease for BC risk, compared with the QQ genotype, resembling the ORs trend obtained for the analysis in the overall population. In contrast, in premenopausal women with local disease no such tendency was observed. Thirty-six percent of cases and 37% of controls carried at least one R allele. In the postmenopausal group, association between PON1 Q192R genotypes and BC risk was found for both local and advanced cases. We found that significant decreases in the frequencies of the R allele occurred for local as well as advanced BC. About 3% of cases and 38% of controls carried at least one R allele in both local and advanced postmenopausal group. By contrast, an increase was observed in the frequencies of the PON1 55 M allele in both local and advanced premenopausal BC groups, with respect to the control cohort, thus suggesting an increased risk of BC. In particular, 85% of cases and 66% of controls were carriers of at least one R allele, in the local premenopausal group, and 82% of cases and 66% of controls were carriers of at least one R allele, in the advanced premenopausal group. A similar trend was observed in postmenopausal women. In particular, individuals with the PON1 55 LM and MM genotypes showed an increased risk of advanced BC. Potential hormone-related BC risk factors (age at menarche, postmenopausal use of estrogens, use of oral contraceptives, parity, age at FFTP, BMI or WHR) did not modify the association of either Q192R or L55M SNPs with BC (data not shown).

**Table 8:** Associations between PON1 Q192R and L55M genotypes and breast cancer

PON1 Q192R	QQ	QR	RR	QR and RR
	
Premenopausal				
Controls, n (%)	144 (62.6)	64 (27.8)	22 (9.6)	86 (37.4)
Cases by stage				
Local, n (%)	70 (63.6)	30 (27.3)	10 (9.1)	40 (36.4)
OR (95% CI)^a^	1.0	0.96 (0.55–1.60)	0.94 (0.32–2.17)^b^	0.95 (0.60–1.62)
Advanced, n (%)	65 (84.4)	11 (14.3)	1 (1.3)	12 (15.6)
OR (95% CI)^a^	1.0	0.42 (0.23–0.86)	0.23 (0.06–0.85)^c^	0.33 (0.14–0.60)
Postmenopausal				
Controls, n (%)	196 (62.4)	88 (28.0)	30 (9.6)	118 (37.6)
Cases by stage				
Local, n (%)	221 (96.9)	6 (2.6)	1 (0.50)	7 (3.1)
OR (95% CI)^a^	1.0	0.06 (0.01–0.12)	0.04 (0.00–0.23)^d^	0.05 (0.00–0.10)
Advanced, n (%)	128 (96.9)	3 (2.3)	1 (0.8)	4 (3.1)
OR (95% CI)^a^	1.0	0.04 (0.01–0.16)	0.02 (0.00–0.25)^e^	0.05 (0.01–0.11)
PON1 L55M	LL	LM	MM	LM and MM
	
Premenopausal				
Controls, n (%)	78 (33.9)	70 (30.4)	82 (35.7)	152 (66.1)
Cases by stage				
Local, n (%)	16 (14.6)	25 (22.7)	69 (62.7)	94 (85.4)
OR (95% CI)^a^	1.0	1.71 (0.78–2.99)	4.12 (2.13–7.98)^f^	3.05 (1.63–5.78)
Advanced, n (%)	14 (18.2)	8 (10.4)	55 (71.4)	63 (81.8)
OR (95% CI)^a^	1.0	0.62 (0.22–1.70)	3.71 (1.80–7.53)^g^	2.28 (1.12–4.49)
Postmenopausal				
Controls, n (%)	110 (35.0)	55 (17.5)	149 (47.5)	204 (65.0)
Cases by stage				
Local, n (%)	49 (21.5)	52 (22.8)	127 (55.7)	179 (78.5)
OR (95% CI)^a^	1.0	2.15 (1.28–3.75)	1.94 (1.26–2.98)^h^	1.88 (1.20–2.59)
Advanced, n (%)	28 (21.2)	30 (22.7)	74 (56.1)	104 (78.8)
OR (95% CI)^a^	1.0	2.16 (1.15–4.22)	1.98 (1.19–3.36)^i^	2.00 (1.18–3.29)

^a^Adjusted for age, age at menarche, age at FFTP, number of full term pregnancies, first-degree family history of breast cancer, history of benign breast disease, use of oral contraceptives, WHR, use of non-steroidal anti-inflammatory drugs (NSAID), alcohol consumption, smoking habits, level of education and postmenopausal use of estrogen. ^b^P for trend = 0.31; ^c^P for trend = 0.02; ^d, e , f, g, h, i^P for trend = 0.003.

### GLO1 polymorphism and BC risk

The distribution of GLO1 genotypes and the ORs associated with BC are shown in Table 9. We found that 78% of cases and 42% of controls were carriers of at least one E allele. When stratified by menopausal status, the frequency of the E allele was higher among both premenopausal (79%) and postmenopausal (78%) cases compared with the respective frequencies (55% and 32%) in the control subjects. Patients with the AE genotype and EE genotype had a significant increased BC risk compared with the AA genotype. Results stratified by menopausal status at diagnosis (Table 9) and according to tumor stage (Table 10) confirmed the ORs of the general analysis. No significant association was found between GLO1 polymorphism and BC risk according to potential hormone-related BC risk factors (age at menarche, postmenopausal use of estrogens, use of oral contraceptives, parity, age at FFTP, BMI or WHR) (data not shown).

**Table 9:** Associations between GLO1 genotype and breast cancer

	AA	AE	EE	AA and AE
	
All				
Cases, n (%)	119 (21.8)	261 (47.7)	167 (30.5)	428 (78.2)
Controls, n (%)	317 (58.3)	159 (29.2)	68 (12.5)	227 (41.7)
OR (95% CI)^a^	1.0	4.32 (3.20–5.87)	6.52 (4.51–9.40)^b^	4.94 (3.75–6.50)
Menopausal status at diagnosis				
Premenopausal				
Cases, n (%)	39 (20.9)	84 (44.9)	64 (34.2)	148 (79.1)
Controls, n (%)	104 (45.2)	88 (38.3)	38 (16.5)	126 (54.8)
OR (95% CI)^a^	1.0	2.50 (1.51–4.18)	4.46 (2.50–7.98)^b^	3.12 (1.96–4.94)
Postmenopausal				
Cases, n (%)	80 (22.2)	177 (49.2)	103 (28.6)	280 (77.8)
Controls, n (%)	213 (67.8)	71 (22.6)	30 (9.6)	101 (32.2)
OR (95% CI)^a^	1.0	6.54 (4.38–9.76)	9.11(5.48–15.05)^b^	7.35 (5.14–10.53)

^a^Adjusted for age, age at menarche, age at FFTP, number of full term pregnancies, first-degree family history of breast cancer, history of benign breast disease, use of oral contraceptives, WHR, alcohol consumption, smoking habits, level of education and postmenopausal use of estrogen. ^b^P for trend: all, 0.01; premenopausal, 0.03; postmenopausal, 0.01.

**Table 10:** Associations between GLO1 genotype and breast cancer according to menopausal status and tumor stage

	AA	AE	EE	AE and EE
	
Premenopausal				
Controls, n (%)	104 (45.2)	88 (38.3)	38 (16.5)	126 (54.8)
Cases by stage				
Local, n (%)	26 (23.6)	50 (45.4)	34 (31.0)	84 (76.4)
OR (95% CI)^a^	1.0	2.17 (1.16–4.0)	3.58 (1.82–7.08)^b^	2.66 (1.58–4.57)
Advanced, n (%)	13 (16.9)	34 (44.1)	30 (39.0)	64 (83.1)
OR (95% CI)^a^	1.0	3.05 (1.35–6.65)	6.29 (2.78–14.34)^c^	4.08 (2.02–4.20)
Postmenopausal				
Controls, n (%)	213 (67.8)	71 (22.6)	30 (9.6)	101 (32.2)
Cases by stage				
Local, n (%)	55 (24.1)	98 (43.0)	75 (32.9)	173 (75.9)
OR (95% CI)^a^	1.0	5.31 (3.39–8.25)	9.64 (5.59–16.82)^d^	6.63 (4.43–9.95)
Advanced, n (%)	25 (19.0)	79 (59.8)	28 (21.2)	107 (81.0)
OR (95% CI)^a^	1.0	9.44 (5.40–16.55)	7.99 (3.95–16.30)^e^	8.66 (4.89–15.20)

^a^Adjusted for age, age at menarche, age at FFTP, number of full term pregnancies, first-degree family history of breast cancer, history of benign breast disease, use of oral contraceptives, WHR, alcohol consumption, smoking habits, level of education and postmenopausal use of estrogen. ^b, c, d, e^P for trend = 0.001.

### Combination of CYP17, GSTP1, PON1-192, PON1-55 and GLO1 polymorphisms as predictive factor for BC risk

The analysis of a single polymorphism alone is often not indicative for the association to the risk of BC. We believe that the combination of them all in each single case may be a more predictive factor for the risk of this neoplasia. Therefore, since these genes are all related to the control of oxidative stress-inducing mechanisms, we would like to emphasize that the detection of the GSTP1IleVal-PON1/192QR-PON1/55LM-GLO1AE or GSTP1IleIle-PON1/192QQ-PON1/55MM-GLO1EE genotype combination at individual level, might lead to the identification of patients with intermediate and high risk for BC, respectively (Table 11), compared to the GSTP1ValVal-PON1/192RR-PON1/55LL-GLO1AA genotype combination.

**Table 11:** Risk's level according to genotypes combination

	Cases	Controls			
				
Genotype	n	n	OR^a^	95% CI	P
Reference genotype	254	633	1.0		
Intermediate risk	643	776	2.32	1.79–2.54	< 0.001
High risk	1291	767	4.22	3.55–5.02	< 0.001

^a^Adjusted for age, age at menarche, age at FFTP, number of full term pregnancies, first-degree family history of breast cancer, history of benign breast disease, use of oral contraceptives, WHR, alcohol consumption, smoking habits, level of education and postmenopausal use of estrogen. ORs are calculated relative to subjects with the reference genotype.

Reference genotype GSTP1ValVal-PON1/192RR-PON1/55LL-GLO1AA.

Intermediate risk: GSTP1IleVal-PON1/192QR-PON1/55LM-GLO1AE.

High risk: GSTP1IleIle-PON1/192QQ-PON1/55MM-GLO1EE.

None of the SNPs was significantly associated with the risk of BC after controlling for multiple testing by Bonferroni analysis (data not shown).

## Discussion

The aetiological factors implicated in breast carcinogenesis are unclear, but estrogen levels [2], lifestyle factors, especially diet [43], oxidative and carbonyl stresses [4-8] have all been suggested to influence breast cancer (BC) risk. The human body has a number of enzyme systems protecting from genotoxic damage, acting either indirectly, via reduction of potential substrates giving free radicals production, such as cytochrome P450c17α (CYP17) [38,39] or, directly, via free radical detoxification, such as glutathione S-transferase (GSTP1), paraoxonase (PON1) [40] and glyoxalase 1 (GLO1) [41]. Polymorphisms of these genes are believed to be key factors in determining cancer susceptibility to toxic or environmental chemicals [29,44,45]. Therefore, in an effort to increase our understanding of the interaction between potential carcinogenic environmental exposure and genetic factors in the pathogenesis and predisposition to BC disease risk, we determined the frequencies and the relative risks (ORs) of CYP17, GSTP1, PON1 and GLO1 gene polymorphisms in a control population and in a population of patients with breast tumor. In agreement with the majority of studies [19-21,23,24] on CYP17 polymorphism and BC risk, our results did not reveal any significant association between the CYP17 A2 allele and the risk of BC. Conversely, the present study revealed that CYP17 polymorphism may lower the risk of BC in premenopausal women with the A1A1 genotype and later age at menarche or with at least one child, in accordance with the results obtained by others [19-21]. Our observations may be compatible with the hypothesis that the protection against BC is reduced among women with the A2 allele containing genotypes, because of elevated baseline levels of circulating steroid hormones. In addition, results from a previous study indicate that nulliparous women with the A2A2 genotype have higher mean levels of serum estradiol than those with the A1A1 genotype [46]. Moreover, the fact that the observed associations were mainly confined to premenopausal women, could reflect differences in the nature of premenopausal and postmenopausal BC etiology [47] and suggest that CYP17 gene polymorphism may play a different role in later onset of the disease.

As to GSTP1, we found that the major risk for BC was associated with individuals homozygotes for the Ile allele, while the Val allele appeared to be a protective factor against BC. A similar trend was presumably related with an activation of the enzyme at tissue level. Besides, experimental evidences showed that the Val variant may have either lower or higher specific activity and affinity depending on the substrate [48]. On this aspect, our findings may differ from those of other studies showing an increased risk for women carrying the Val allele, due to different environmental exposure and/or dietary habits. Our results are instead consistent with other studies where a tendency of decreased risk could be seen for the GSTP1 Val allele [25,26]. As well known, oxidative stress and free radicals induced by environmental and/or endogenous carcinogens have been associated with increased risk of BC [4-6].

Regarding PON1, we found that women with PON1-192/QR and RR genotype had a lower risk of BC in comparison to women with the PON1-192/QQ genotype. This might be explained since, as expected, the Q to R substitution lead to the production of an enzyme with a higher detoxification activity against potentially carcinogenic products of oxidative stress and lipid peroxidation. This is supported by a quite recent study showing that individuals with a higher PON1 activity had a higher frequency of genotypes containing the R and L alleles [28,49]. When the analysis was carried out in subgroups of women according to their menopausal status at diagnosis, the PON 192R variant was associated with a decreased risk of developing BC only among postmenopausal women. Similarly, the PON 192R variant was associated with a decreased risk of BC only among premenopausal women with advanced BC and among postmenopausal patients with both local and advanced BC when compared to controls. As for CYP17 SNP, our results suggest that also PON1 Q192R polymorphism may play a different role in premenopausal and postmenopausal BC etiology. In addition, exogenous factors, environmental conditions, dietary habits and life-style other than genetic components alone may play a role in determining paraoxonase enzyme activity. Moreover, findings from the present study about the association of PON1-192R variant and BC risk are consistent with those of Gallicchio et al. [33]. On the contrary, with regard to L55M SNP, our data are in agreement with those of Stevens et al. [34].

Regarding GLO1 polymorphism, our results indicate that the presence of the GLO1 E allele significantly increases BC risk. This might be explained by the fact that the A to E substitution, due to the SNP, may determine a conformational modification in the enzyme, leading to an isoenzyme with a lower detoxification capacity [36]. As well known, glyoxalase 1 is an efficient antiglycation defence that decreases the concentration of reactive carbonyl compounds, such as methylglyoxal (MG), one of the most potent precursors of carbonyl stress-related advanced glycation end-products (AGEs). Consequently, a decrease in the activity of this enzyme may result in an accumulation of AGEs in human breast tissues. Since AGEs, resulting from sugar-derived protein modifications, are known to play a role in BC pathogenesis and progression [7,8], it would be expected that the presence of a lower activity form of GLO1 – the GLO1 allele – may be predictive about severe consequences for an individual's BC risk. Indeed, accumulations of AGEs in the serum of BC patients [7,8] has been recently described. In addition, we found that the risk of BC associated with the GLO1 E allele significantly tended to increase according to tumour stage, thus suggesting a possible role of this polymorphism, not only in the development of this neoplasia but also in progression of local to advanced BC. Since this is the first study to examine the association between GLO1 A111E polymorphism and the risk of BC, additional research is required to confirm these findings.

The single SNP associations found in this study did not withstand correction for multiple testing by Bonferroni method. Additional data are therefore needed to corroborate our findings. These may be derived by replication in other genetic-epidemiological studies, preferably in combination with studies on the functional role of gene products that provide biological plausibility.

We also determined the genotypic and allelic frequencies of CYP17, GSTP1, PON1-192 and-55 as well as GLO1 polymorphisms for each considered group (control and patients). When tested for HWE, the genotypic frequencies were in agreement with those predicted under HWE only for CYP17 polymorphism. Conversely, there were statistically significant deviations among the controls between the observed and expected genotypic frequencies for all the other polymorphisms. Such deviations, in literature, have been attributed primarily to a possibility of genotyping error [50]. We have ruled out this possibility by inclusion of samples of known genotypes, as positive RFLP controls and three independent operators confirmed genotypes. In addition, the frequencies of the mutant allele among controls for most of the SNPs under consideration were not significantly different from those one observed among Caucasian women in other published studies [20,33,51]. Other possible reasons for the observed deviations could be either genetic drift, migration, inbreeding, cultural parameters based on religion beliefs and socio-economic requirements, recent origin/introduction of the polymorphism, absence of random mating, and/or a stratification bias. The last possibility can be ruled out since our sample collection included individuals of known ethnicity. However, the other factors cannot be ruled out, since our current sample set is not appropriate to comment on the selection forces operating on the population.

## Conclusion

In conclusion, the results of this report suggest that polymorphisms of genes involved in the oxidative stress control may play a significant role in the development of BC. In particular, CYP17 polymorphism may modify the risk of BC only for certain subgroups, while GSTP1, PON1-192, PON1-55 and GLO1 polymorphisms appear to be common genetic traits likely associated with the risk of BC. However, the results of our exploratory analysis, while nominally significant, did not withstand correction for multiple testing. Therefore, further studies are needed to confirm our findings and to explore the exact molecular basis of our observations.

## Abbreviations

BC: Breast cancer; CYP17: Cytochrome P450C17α; GSTP1: Glutathione S-transferase; PON1: Paraoxonase 1; GLO1: Glyoxalase 1; PCR/RFLP: Polymerase Chain Reaction/Restriction fragment length polymorphism; ROS: Reactive oxygen species; LDL: Low density lipoproteins; AGEs: Advanced glycation end-products; MG: Methylglyoxal; HDL: High density lipoproteins; SNP: Single nucleotide polymorphism; OR: Odds ratio; CI: Confidence intervals; FFTP: First full-term pregnancy; WHR: Waist-hip ratio; BMI: Body mass index; SD: Standard deviation.

## Competing interests

The authors declare that they have no competing interests.

## Authors' contributions

CA participated in the design of the study and its coordination. Performed the statistical analysis and drafted the manuscript. CDB carried out the burden of the molecular assays (PCRs and RFLP). VL and SG participated in the design of the study and in carrying out the initial phases of the molecular assays (DNA extraction). AR and FB participated in the design of the study and recruited all patients and collected all clinical data. VT conceived of the study and helped to draft the manuscript. All authors read and approved the final manuscript.

## Pre-publication history

The pre-publication history for this paper can be accessed here:

http://www.biomedcentral.com/1471-2407/9/115/prepub
